# Probe Selection and Expression Index Computation of Affymetrix Exon Arrays

**DOI:** 10.1371/journal.pone.0000088

**Published:** 2006-12-20

**Authors:** Yi Xing, Karen Kapur, Wing Hung Wong

**Affiliations:** 1 Department of Statistics, Stanford University Stanford, California, United States of America; 2 Department of Internal Medicine, Roy J. and Lucille A. Carver College of Medicine, University of Iowa Iowa City, Iowa, United States of America; Max Planck Institute for Evolutionary Anthropology, Germany

## Abstract

**Background:**

There is great current interest in developing microarray platforms for measuring mRNA abundance at both gene level and exon level. The Affymetrix Exon Array is a new high-density gene expression microarray platform, with over six million probes targeting all annotated and predicted exons in a genome. An important question for the analysis of exon array data is how to compute overall gene expression indexes. Because of the complexity of the design of exon array probes, this problem is different in nature from summarizing gene-level expression from traditional 3′ expression arrays.

**Methodology/Principal Findings:**

In this manuscript, we use exon array data from 11 human tissues to study methods for computing gene-level expression. We showed that for most genes there is a subset of exon array probes having highly correlated intensities across multiple samples. We suggest that these probes could be used as reliable indicators of overall gene expression levels. We developed a probe selection algorithm to select such a subset of highly correlated probes for each gene, and computed gene expression indexes using the selected probes.

**Conclusions/Significance:**

Our results demonstrate that probe selection improves gene expression estimates from exon arrays. The selected probes can be used in future analyses of other exon array datasets to compute gene expression indexes.

## Introduction

Microarrays have become one of the most popular technologies for profiling gene expression since its invention more than a decade ago [1]–[3]. Expression microarrays use probes targeting specific genes based on nucleotide sequence complementarity to quantitatively measure mRNA levels for tens of thousands of genes. A variety of gene expression microarray platforms are used today, including spotted cDNA arrays, Affymetrix GeneChip arrays, Agilent ink-jet arrays and Illumina long-oligonucleotide bead-based arrays [4], [5]. These microarray platforms differ in their probe design, hybridization protocol, labeling and production methods [4], [5]. Despite their differences, traditional gene expression microarray platforms share a common goal–obtaining a single value for each gene representing its overall mRNA abundance in a given sample. For example, the traditional Affymetrix GeneChips use one or more probesets consisting of 11 perfect-match (PM) and 11 mismatch (MM) probes targeting the 3′ end of the mRNA sequence. The signals from multiple probes are summarized into a single value as the gene expression index [6]. Throughout this manuscript we will refer to the traditional Affymetrix GeneChips, such as the Affymetrix human U133-PLUS2 arrays as the 3′ expression arrays.

Recently, global analyses of mammalian transcriptomes suggest that alternative splicing is an important and prevalent form of transcript variation in many species [7]. Alternative splicing refers to the production of multiple transcript isoforms from a single gene, due to variations in pre-mRNA splicing [8]. Genome-wide analyses of expressed sequences indicate that 40–60% of human genes have multiple splice forms [9]. Since alternative splicing has been largely ignored throughout the probe design of traditional expression microarrays, these findings have motivated the development of a new generation of microarray platforms, which use probes targeting individual exons to interrogate pre-mRNA splicing at the genomic scale [10]–[14].

**Figure 1:**
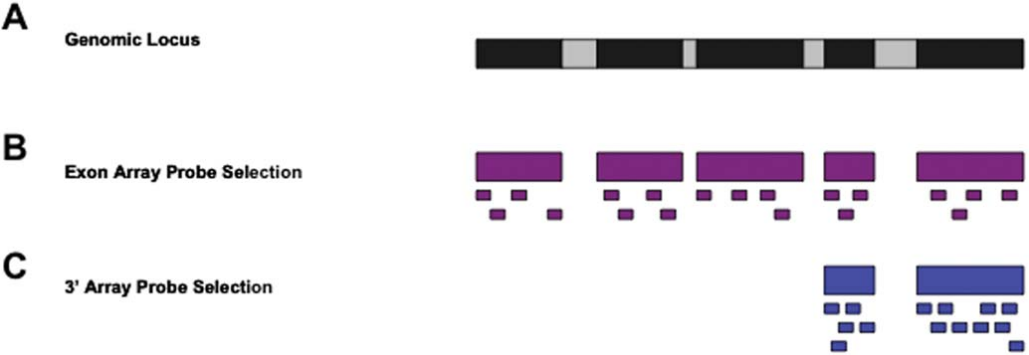
Probe design of exon arrays. (**A**) Exon-intron structure of a gene. Black boxes represent exons. Gray boxes represent introns. Introns are not drawn to scale. (**B**) Probe design of exon arrays. Exon arrays have four probes targeting each exon of the gene. (**C**) Probe design of 3′ expression arrays. Probes on 3′ expression arrays target 3′ end of the mRNA sequence.

Last year, Affymetrix released ‘exon arrays’, a high-density microarray platform with a total of ∼6.5 million probes targeting all the annotated and predicted exons in the genome. Exon arrays differ significantly from 3′ expression arrays in the number and placement of the oligonucleotide probes. In the 3′ expression arrays a probeset consisting of 11 probes is selected from the 3′ end of the mRNA sequence. In contrast, in exon arrays 4 probes are selected from each putative exonic region (Figure 1, modified from Affymetrix Exon Array design datasheet [15]). Many genes have more than a hundred probes on the exon array. Exon array probesets are classified based on the level of annotational confidence. Briefly, probes targeting exons with RefSeq mRNA evidence are regarded as the most confident and are referred to as “core probes”. Probes targeting exons with EST evidence are referred to as “extended probes”. Probes targeting putative computational exon predictions have the least confidence and are referred to as “full probes”. For further details, see the Affymetrix technical documentation for Exon Array probe annotations [16].

An important question for the analysis of exon array data is how to compute overall gene expression indexes. In the context of exon array analyses, we define the overall gene expression index as the total abundance of all molecules transcribed from a single gene, including various alternative splice forms, alternative 5′ transcripts, alternative poly A transcripts, etc. The whole-transcriptome amplification protocol and probe design of exon arrays allow a more precise monitoring of such overall gene expression indexes. The gene-level expression estimates from Exon Arrays can be used in standard high-level analyses of microarrays (such as clustering). They also provide the basis for subsequent analyses of RNA alternative splicing [17]. However, because of the complexity of the design of exon array probes, this problem is different in nature from summarizing gene-level expression from traditional 3′ expression arrays. In this manuscript, we present our probe-level analysis of the exon array data, and describe a probe selection strategy for computing gene expression indexes from Exon Arrays.

## Materials and Methods

### Processing of probe-level data

We downloaded the public Human Exon 1.0 ST Array tissue panel dataset (http://www.affymetrix.com/support/technical/sample_data/exon_array_data.affx) consisting of 11 human tissues (breast, cerebellum, heart, kidney, liver, muscle, pancreas, prostate, spleen, testes, and thyroid) each with three replicates. We normalized the data by sketch normalization using the Affymetrix Exon Array Computational Tool (ExACT) (http://www.affymetrix.com/products/software/specific/exact.affx). For each gene (referred to as a ‘transcript cluster’ on the exon array platform), we calculated the Pearson correlation coefficient of the signal intensities of all possible pairs of probes across the 11 tissues (a total of 33 samples). We visualized the correlation matrix of the probe intensities as a heatmap using R (http:///www.r-project.org).

### Probe selection algorithm

In this section, we describe an algorithm to automatically select a subset of core probes for each gene as the reliable indicators of overall gene expression. Although alternative splicing is prevalent in the human genome and occurs in nearly three quarters of multi-exon human genes, within each gene the majority of exons are still constitutively spliced [18]. We reason that the majority of probes targeting those constitutive exons show correlated intensities across various human tissues, and reflect the overall mRNA abundance of the gene. Therefore, our algorithm seeks to identify the largest subset of highly correlated probes.

Because the traditional Affymetrix GeneChips had 11 perfect-match probes in each probeset of a gene, we decided to select at least 11 probes for each gene on the exon array. If a gene had less than 11 core probes, we skipped probe selection and simply used all the core probes for computing gene expression indexes. The probe selection procedure is summarized below. Briefly, we calculated the Pearson correlation coefficient of the signal intensities of all probe pairs across the 11 tissues (a total of 33 samples). Using (1-Pearson correlation coefficient) as the distance metric, we performed average-linkage hierarchical clustering for all core probes. Next, we cut the clustering dendrogram at different heights *h* = (0.1, 0.2, 0.3…1.0), and calculated the size of the biggest subcluster under each cutoff, excluding probes from probesets with only one probe in the biggest subcluster. We chose a cutoff to achieve a balance between the size of the biggest subcluster and the average correlation within the biggest subcluster.

Exon Array probe selection algorithm:

- If the number of core probes is less than 11 for a transcript cluster, we select all the core probes.
- If the number of core probes is greater than 11:
-
  - We apply hierarchical clustering to the 11-tissue data for all core probes (distance metric: 1-Pearson correlation; average linkage clustering). We cut the clustering dendrogram at various heights *h* = (0.1, 0.2, 0.3…1.0) and calculate the size (*S*) of the biggest subcluster at each cutoff, excluding probesets with only 1 probe in the biggest cluster. A small *h* means that we cut the clustering dendrogram near its bottom, while a large *h* means that we cut the clustering dendrogram near its top. We choose the smallest *h* (*h*_0_) with the corresponding *S* (*S*_0_)> = 11.
  - If *h*_0_> = 0.5, we choose *h_final_* = *h*_0_.
  - If *h*_0_< = 0.4, we choose the *h_final_* from *h*_0_, *h*_0_+0.1…0.4 to maximize 
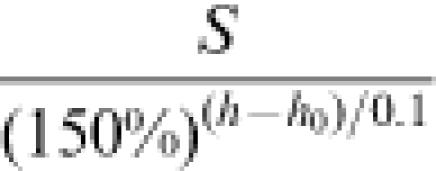
. Intuitively, this means if we want to increase the tree cutoff by 0.1, the size of the biggest subcluster needs to increase by at least 50%. We chose this number (50%) from our empirical analysis of exon array data in a number of genes.
  - Cut the clustering dendrogram at *h_final_*, and select probes in the biggest subcluster, excluding probesets with only one core probe in the biggest subcluster.

### Gene expression index computation

We computed the gene expression indexes for each gene across the 11 human tissues (a total of 33 samples), by fitting the probe level data to the Li-Wong model [6], implemented in the affy package of Bioconductor (http://www.bioconductor.org/).

We compiled a list of genes differentially expressed between liver and muscle based on 3′ expression array data. We downloaded the Affymetrix human U133-PLUS2 array dataset on the same tissue samples (http://www.affymetrix.com/support/technical/sample_data/exon_array_data.affx). We normalized the data (quantile-normalization) and computed the gene expression indexes (Li-Wong model [6]) using dChip (http://biosun1.harvard.edu/complab/dchip/). To select differentially expressed genes, we required a probeset to have a fold change of at least 5, and an absolute difference in gene expression indexes of at least 200. We matched human U133-PLUS2 probesets and human Exon Array transcript clusters using the mapping provided by Affymetrix.

## Results

### Visualization of the probe-level data

**Figure 2:**
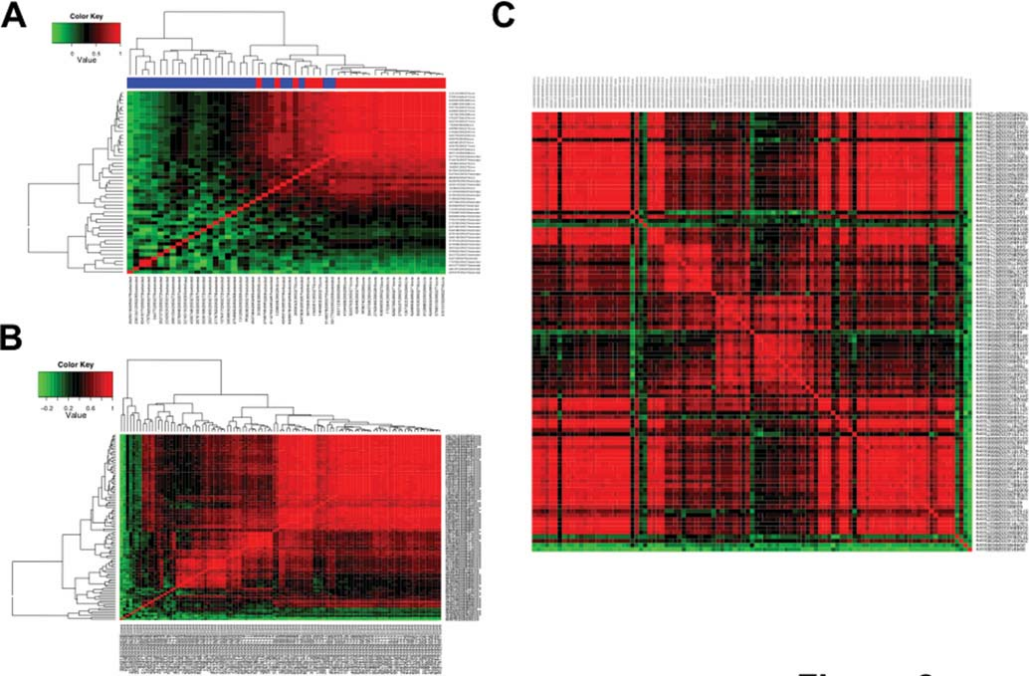
Heatmap visualization of exon array pairwise probe correlations. (**A**) Heatmap visualization of probe intensities of HLA-DMB (transcript cluster 2950263). Each cell of the heatmap shows the correlation of two probes in 11 tissues. The top color bar indicates the probe type. Core probes are colored in red. Extended probes are colored in blue. The signal intensities of core probes usually have a high correlation (the top right corner of the heatmap). (**B**) Heatmap visualization of probe intensities of core probes in CD44 (transcript cluster 3326635). Probes targeting the 5′ and 3′ regions (constitutive exons) of CD44 show highly correlated signals in 11 tissues (the top right corner of the heatmap). (**C**) Heatmap visualization of probe intensities of core probes in CD44 (transcript cluster 3326635). Probes are ordered from top to bottom based on their genomic coordinates (5′ to 3′).

Our heatmap visualization of the probe-level data reveals interesting patterns. Figure 2 shows the heatmap plotted for two human genes, each with well-annotated gene structure and/or patterns of alternative splicing. Each cell of the heatmap reflects the Pearson correlation coefficient of two probes' intensities in 33 samples. In Figure 2A, we visualize the entire correlation matrix for all probes belonging to the gene HLA-DMB (transcript cluster 2950263), including 24 core probes and 28 extended probes. HLA-DMB has six exons and plays an important role in class II antigen presentation [19]. From the heatmap, it is apparent that about half of the probes of HLA-DMB are highly correlated with each other across the 11 human tissues (top right corner of the correlation matrix), while the remaining probes are very poorly correlated with other probes of this gene. It is interesting to note that the vast majority of core probes are located in the top right corner of the heatmap, suggesting that their intensities change coordinately across different samples. In contrast, most of the extended probes show poor correlation with each other and with the core probes, suggesting that they are mostly reflecting background noise and are poor indicators of overall gene expression. This is a typical pattern (i.e. core probes tend to be correlated) for the majority of genes on the exon array. In addition, core probes usually have much stronger signals than extended and full probes, consistent with the design that core probes target high-confidence exons in the genome. Taken together, these data suggest that extended and full probes are usually poor indicators of overall gene expression.

Can we simply take all the core probes of a gene to compute its expression indexes? The answer is no. Some core probes have low affinity and their intensities are largely saturated by background noise. Some core probes cross-hybridize to other gene targets. Some core probes target alternatively spliced regions of the genes. These probes are not good indicators of overall gene expression. To illustrate such effects, Figure 2B shows the correlation matrix of core probes of CD44 (transcript cluster 3326635). CD44 has 20 exons and its alternative splicing pattern has been well characterized [20], [21]. The exons in the 5′ and 3′ regions of CD44 are constitutively spliced, while ten exons in the middle of the gene undergo extensive alternative splicing in various tissues and cancers. The heatmap (see Figure 2B) indicates a large group of probes with highly correlated intensities (the top right corner of the heatmap). There is a second group of correlated probes (between the center and the bottom left corner of the heatmap) with weaker correlation with the first group. Checking the exon array probe annotation through the Integrated Genome Browser (http://www.affymetrix.com/support/developer/tools/download_igb.affx), we saw that the first group had probes targeting constitutively exons at the 5′ and 3′ regions of CD44, while the second group had probes targeting the alternative exons in the middle of CD44 transcripts (also see Figure 2C, in which CD44 probes were ordered based on their genomic coordinates). In addition, a few core probes (at the bottom left corner of the heatmap) are poorly correlated with all other core probes. Based on the probe-level data and our knowledge of CD44 alternative splicing, probes in the top right corner of the heatmap should be used for summarizing overall gene expression indexes.

Our exploration of the probe-level exon array data indicates the existence of a subset of exon array probes with highly correlated signals in multiple human tissues. These probes tend to have the highest annotational confidence (i.e. core probes) and target the constitutive exons of a gene.

### Probe selection using the human tissue-panel dataset

**Figure 3:**
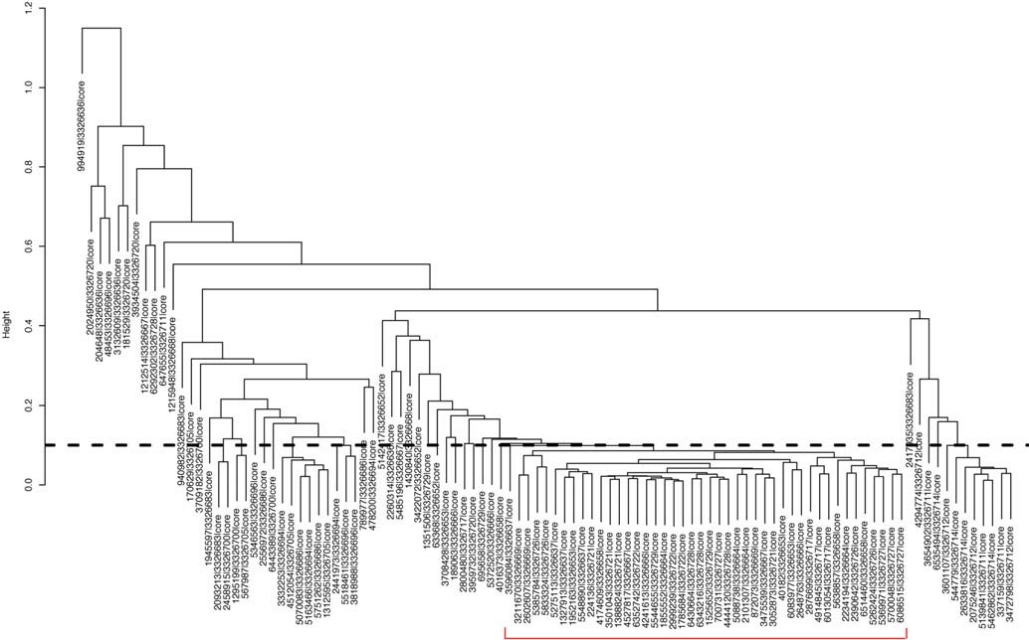
Hierarchical clustering of probe intensities of CD44 core probes. Core probes of CD44 are clustered by average linkage hierarchical clustering based on their intensities in 11 tissues (a total of 33 samples). The distance metric is (1-Pearson correlation coefficient). A total of 44 core probes are selected when we cut the clustering dendrogram at *h* = 0.1 (indicated by the dashed horizontal line).

We used our probe selection algorithm (see Materials and Methods) to select probes from the 11-tissue exon array data. For the reasons stated in the previous section, we applied our probe selection algorithm to core probes of each gene. Using this procedure, we selected 44 core probes for CD44 (42% of its core probes), at the tree cutoff (*h_final_*) of 0.1 (see Figure 3). These 44 core probes targeted the constitutive exons at the 5′ and 3′ ends of the CD44 transcripts. Core probes targeting middle exons of CD44 were not selected.

We applied our probe selection algorithm to 17056 transcript clusters with at least 11 core probes on the human Exon Array. Over the entire dataset, we selected 47.1% of core probes. On average, we selected 26.5 core probes for each transcript cluster. Considering the number of probes for each gene on the traditional Affymetrix GeneChips (typically 11 perfect-match probes), the selected exon array probes using our algorithm should be sufficient for computing gene expression indexes.

### Effects of probe selection on gene expression index estimation

**Figure 4:**
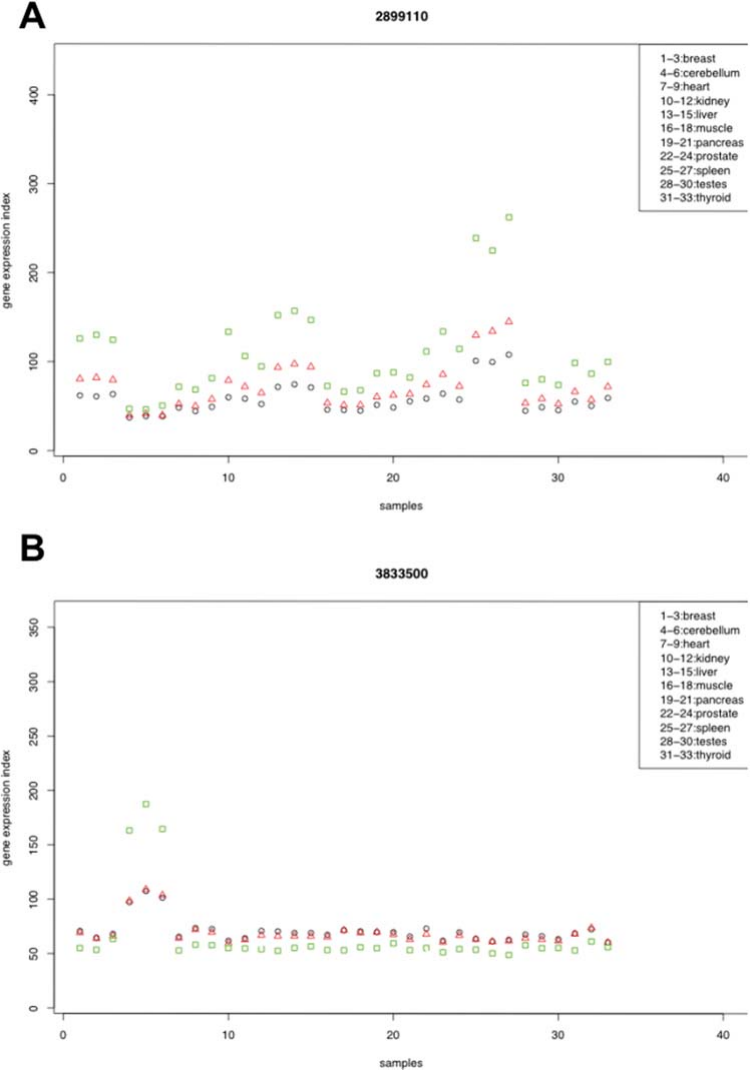
Gene expression indexes computed using all probes, all core probes, and selected core probes. (**A**) Gene expression indexes of transcript cluster 2899110 (HFE) are computed using all probes (black circles), all core probes (red triangles) and selected probes (green rectangles). Probe selection increases the gene expression indexes computed for all the samples. The relative expression levels in different tissues remain unaltered. (**B**) Gene expression indexes of transcript cluster 3833500 (SPTBN4, a gene known to be overexpressed in brain and cerebellum). Probe selection strengthens the pattern of overexpression in cerebellum (sample #4, #5 and #6).

We computed the gene expression indexes for each gene across the 11 human tissues (a total of 33 samples), by fitting the probe level data to the Li-Wong model [6] implemented in the *affy* package of Bioconductor (http://www.bioconductor.org/). We used probe level data from three different sets of probes: all probes, all core probes, or selected core probes. Our analysis shows that including all the probes to compute the gene expression indexes often results in low gene expression estimates across all the samples. This is due to the fact that the vast majority of extended and full probes don't target real exons and have very weak signals on the arrays. To examine the effect of our probe selection, we compared the gene expression indexes computed from all core probes and selected core probes. Figure 4 shows two typical effects of probe selection. In many transcript clusters such as 2899110 (HFE, a gene important in iron metabolism) shown in Figure 4A, probe selection increased the absolute values of gene expression indexes (green rectangles vs red triangles) in each sample. This was expected, because high-affinity probes were less affected by background noise and were more likely to show correlated intensities. Therefore, such probes were more likely to be selected by our probe selection algorithm, increasing the estimated gene expression indexes. Interestingly, in some genes probe selection altered relative gene expression levels among tissues. Figure 4B shows the gene expression indexes computed for beta-spectrin 4 (SPTBN4, transcript cluster 3833500), a gene known to be overexpressed in brain and cerebellum [22]. The gene expression indexes computed from the selected probes strengthened the pattern of over-expression in cerebellum samples.

**Figure 5:**
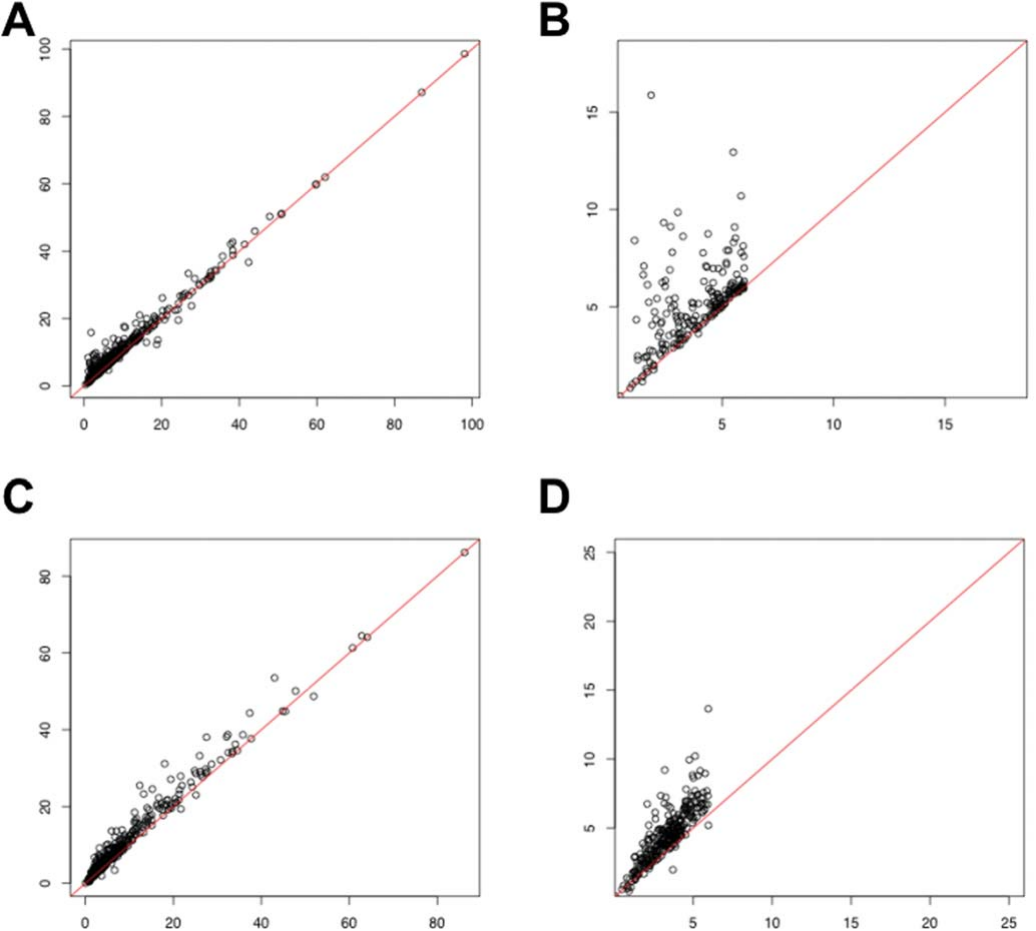
Effects of probe selection on genes differentially expressed between liver and muscle. (**A**) 438 transcript clusters were defined as overexpressed in liver relative to muscle, based on 3′ expression array data (see Materials and Methods). Using gene expression indexes computed from all core probes or the selected core probes, we calculated the average gene expression fold change in three liver samples over three muscle samples. The X-axis shows the fold change using all core probes, and the Y-axis shows the fold change using selected core probes. The red line indicates the 45-degree line (Y = X). (**B**) A magnification of (A) when the fold change computed from all core probes was less than 6. (**C**) 500 transcript clusters were defined as overexpressed in muscle relative to liver, based on 3′ expression array data (see Materials and Methods). Using gene expression indexes computed from all core probes or the selected core probes, we calculated the average gene expression fold change in three muscle samples over three liver samples. The X-axis shows the fold change using all core probes, and the Y-axis shows the fold change using selected core probes. The red line indicates the 45-degree line (Y = X). (**D**) A magnification of (C) when the fold change computed from all core probes was less than 6.

To assess the second effect systematically, we compiled a list of genes differentially expressed between liver and muscle based on 3′ expression array data (see Materials and Methods). Although false positives may be present, the majority of genes in this list should be truly differentially expressed between these two tissues. For 438 transcript clusters overexpressed in liver relative to muscle, we used the exon array data to calculate the average gene expression fold change in three liver samples over three muscle samples. A scatter plot of the fold change shows that our probe selection procedure increased the absolute fold change between liver and muscle (Figure 5A), especially for genes whose original fold change values (without probe selection) were low (Figure 5B). We observed the same trend when we analyzed 500 transcript clusters overexpressed in muscle relative to liver (Figure 5C and 5D). We repeated our analysis in a pairwise comparison of cerebellum and heart samples, and observed a similar trend (data not shown). Therefore, our analysis suggests that we can detect differential gene expression more sensitively using the selected probes from the exon arrays.

### Probe selection is more reliable when gene expression levels are high

**Figure 6:**
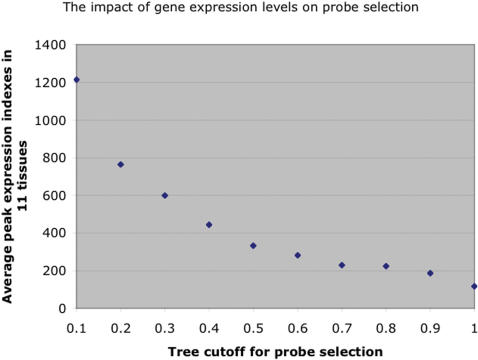
The impact of gene expression levels on probe selection. We grouped 17056 transcript clusters into ten distinct bins according to the height where we cut the clustering dendrogram (*h_final*). For each transcript cluster, we calculated its peak gene expression index in the 11 tissues using selected probes. Then we took an average for each bin. The values of tree cutoff (X-axis) were negatively correlated with average peak gene expression levels in the 11 tissues (Y-axis).

The reliability of probe selection was affected by gene expression levels in these 11 tissues. For 11485 genes, we selected the probes by cutting the clustering dendrogram near its bottom (*h_final*< = 0.4, see the definition of *h_final* in Materials and Methods). These genes tend to have high expression levels in some of the 11 tissues (see Table 1). In contrast, for 5571 genes whose *h_final*> = 0.5, their expression levels in the 11 tissues were much lower (see Table 1). We further grouped 17056 transcript clusters into ten distinct bins according to the height where we cut the clustering dendrogram (*h_final*). The values of *h_final* were negatively correlated with gene expression levels in the 11 tissues (see Figure 6). Our analysis suggests that the result of probe selection was less reliable for lowly expressed genes in the 11 tissues, since we had to cut near the top of the clustering dendrogram to obtain enough selected probes. One explanation is that the signals of most probes of lowly expressed genes are saturated by background or random noise on the arrays, making it difficult to obtain a large group of probes with coordinated changes. For such genes, running probe selection on data from more diverse (e.g. embryonic) cell types (where the genes might be highly expressed) will improve the reliability and reproducibility of selected probes.

**Table 1:**
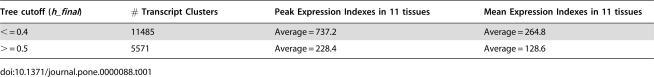
The impact of gene expression levels on probe selection

Tree cutoff (*h_final*)	# Transcript Clusters	Peak Expression Indexes in 11 tissues	Mean Expression Indexes in 11 tissues
< = 0.4	11485	Average = 737.2	Average = 264.8
> = 0.5	5571	Average = 228.4	Average = 128.6

## Discussion

It has been widely realized that not all probes on the microarray are good indicators of overall gene expression [6], [23]. Selecting good probes for robust estimates of gene expression indexes is a common practice in the analysis of oligonucleotide microarray data [6], [23]–[25]. For example, dChip uses a model-based approach to detect one or several outlier probes from the 3′ expression arrays, and excludes those outlier probes from subsequent analyses [6]. Such a simple probe selection strategy, however, might be insufficient for the analysis of exon array data, considering the huge increase in the number of probes, as well as the complexity of these probes' target regions (high-confidence exons, exons only supported by ESTs, computationally predicted exons, etc). Our analysis of the exon array probe level data demonstrates that most extended and full probes have weak signals and are poor indicators of overall gene expression. Even within the core probes, there are probes that cross-hybridize or target alternatively spliced regions of a gene. Sometimes more than half of the core probes are not reliable indicators of overall gene expression (e.g. in the example of CD44, see Figure 2B). A new strategy of probe selection is needed for exon array data analysis.

In this manuscript, we propose a novel probe selection algorithm for the Affymetrix Exon Arrays. We used our method to choose a subset of highly correlated core probes from a public exon array dataset on 11 human tissues. Our analysis of differentially expressed genes among human tissues suggests that probe selection enables a more sensitive detection of gene expression differences. This effect is most prominent when the estimated fold change based on all core probes is low (see Figure 5B and 5D).

We want to emphasize that our probe selection algorithm is a general method that can be adjusted in many flexible ways. For example, for genes with low expression levels in the 11 tissues, our probe selection produced less reliable results. For such genes, it is favorable to run the probe selection algorithm on a larger dataset including samples from other tissues or developmental stages. Another possible adjustment is to consider other types of probes (extended and full probes) in our probe selection procedure. This will be particularly useful if we want to calculate gene expression indexes for poorly annotated or computationally predicted genes on the exon array (which lack core probes). It's also possible to use a model-based approach to iteratively select probes until we reach a fixed number (e.g. 11), such as the IterPLIER algorithm proposed by Affymetrix [26]. Finally, it will be valuable to have a probe selection tool that integrates microarray data and sequence data (such as ESTs). However, this is a non-trivial problem, because sequence data such as ESTs contain various artifacts [9], [27] and are poor indicators of differential splicing [28].

Our selected probes can assist future analyses of other exon array datasets. For 11485 genes, we selected probes from the bottom of the clustering dendrogram from the 11-tissue dataset (see Table 1). These selected probes may already be reliable enough for inference of gene-level expression in new experiments. Probe selection on the remaining genes may await data from more diverse (e.g. embryonic) cell types. In fact, by combining the Affymetrix tissue-panel data with our in-house exon array data on human embryonic stem cells, we could achieve reliable probe selection for 14077 transcript clusters (data not shown). For investigators with a large set of exon arrays, an alternative is to use our algorithm to detect reliable probes based on their own data. It's also possible to combine their own data with other public data (such as the Affymetrix tissue panel) before running probe selection. The gene expression indexes computed from selected probes can be used in standard high-level analyses of expression data, such as clustering, differential expression detection, etc. They also provide a more accurate “baseline” for subsequent analysis of RNA alternative splicing from Exon Arrays.

## Supporting Information

Figure S1 Heatmap visualization (high-resolution) of probe intensities of HLA-DMB (transcript cluster 2950263). Each cell of the heatmap shows the correlation of two probes in 11 tissues. The top color bar indicates the probe type. Core probes are colored in red. Extended probes are colored in blue. The signal intensities of core probes usually have a high correlation (the top right corner of the heatmap).
(0.09 MB PDF)

Figure S2 Heatmap visualization (high-resolution) of probe intensities of core probes in CD44 (transcript cluster 3326635). Probes targeting the 5' and 3' regions (constitutive exons) of CD44 show highly correlated signals in 11 tissues (the top right corner of the heatmap).
(0.28 MB PDF)

Figure S3 Heatmap visualization (high-resolution) of probe intensities of core probes in CD44 (transcript cluster 3326635). Probes are ordered from top to bottom based on their genomic coordinates (5' to 3').
(0.05 MB PDF)
