# Supplementary figures and images for: Probe Selection and Expression Index Computation of Affymetrix Exon Arrays
Source: PLoS One. 2006 Dec 20;1(1):e88. doi: 10.1371/journal.pone.0000088 (PMC1762343; doi:10.1371/journal.pone.0000088)

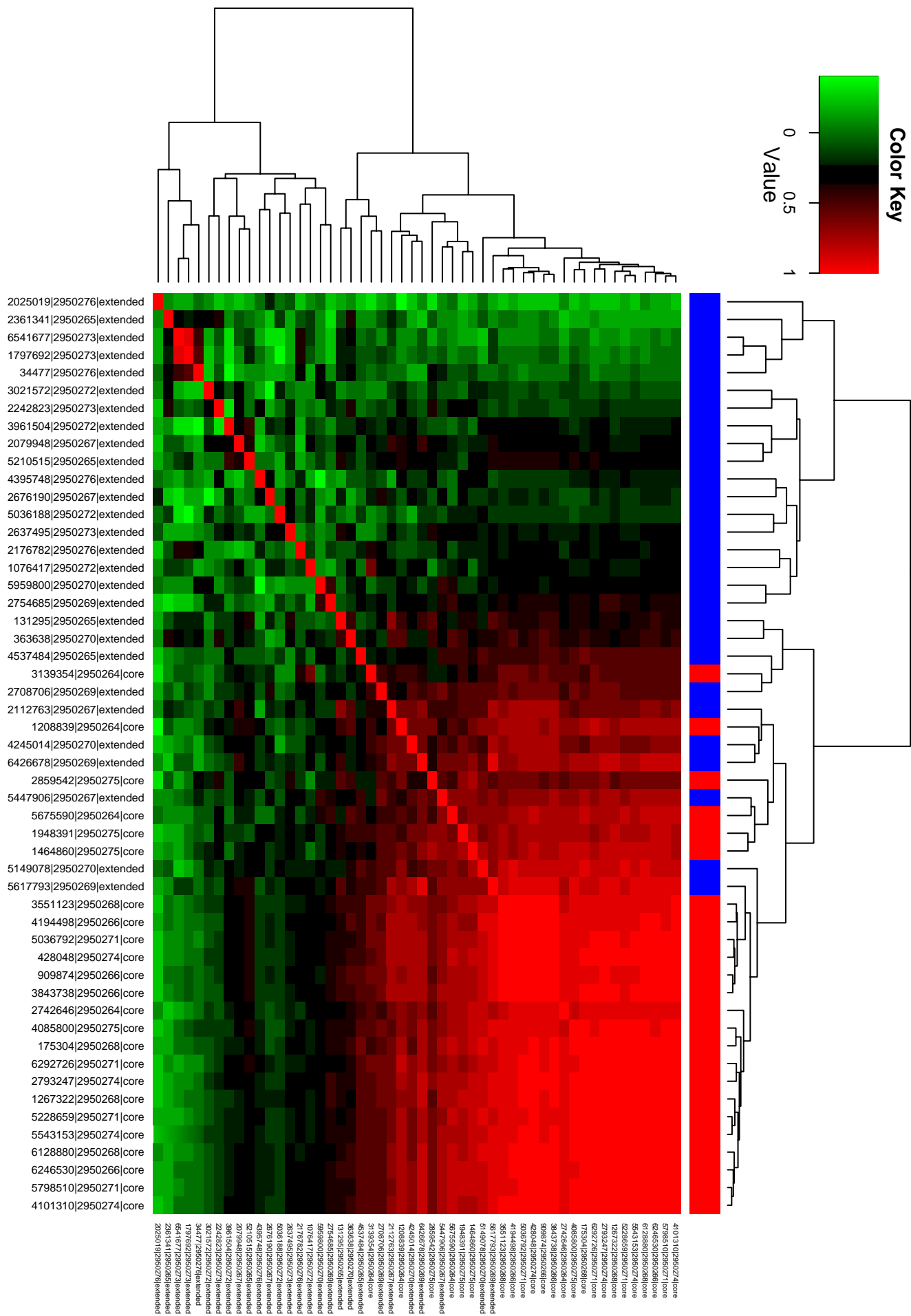

Supplement: Figure S1 — Heatmap visualization (high-resolution) of probe intensities of HLA-DMB (transcript cluster 2950263). Each cell of the heatmap shows the correlation of two probes in 11 tissues. The top color bar indicates the probe type. Core probes are colored in red. Extended probes are colored in blue. The signal intensities of core probes usually have a high correlation (the top right corner of the heatmap). (0.09 MB PDF) [file pone.0000088.s001.pdf]

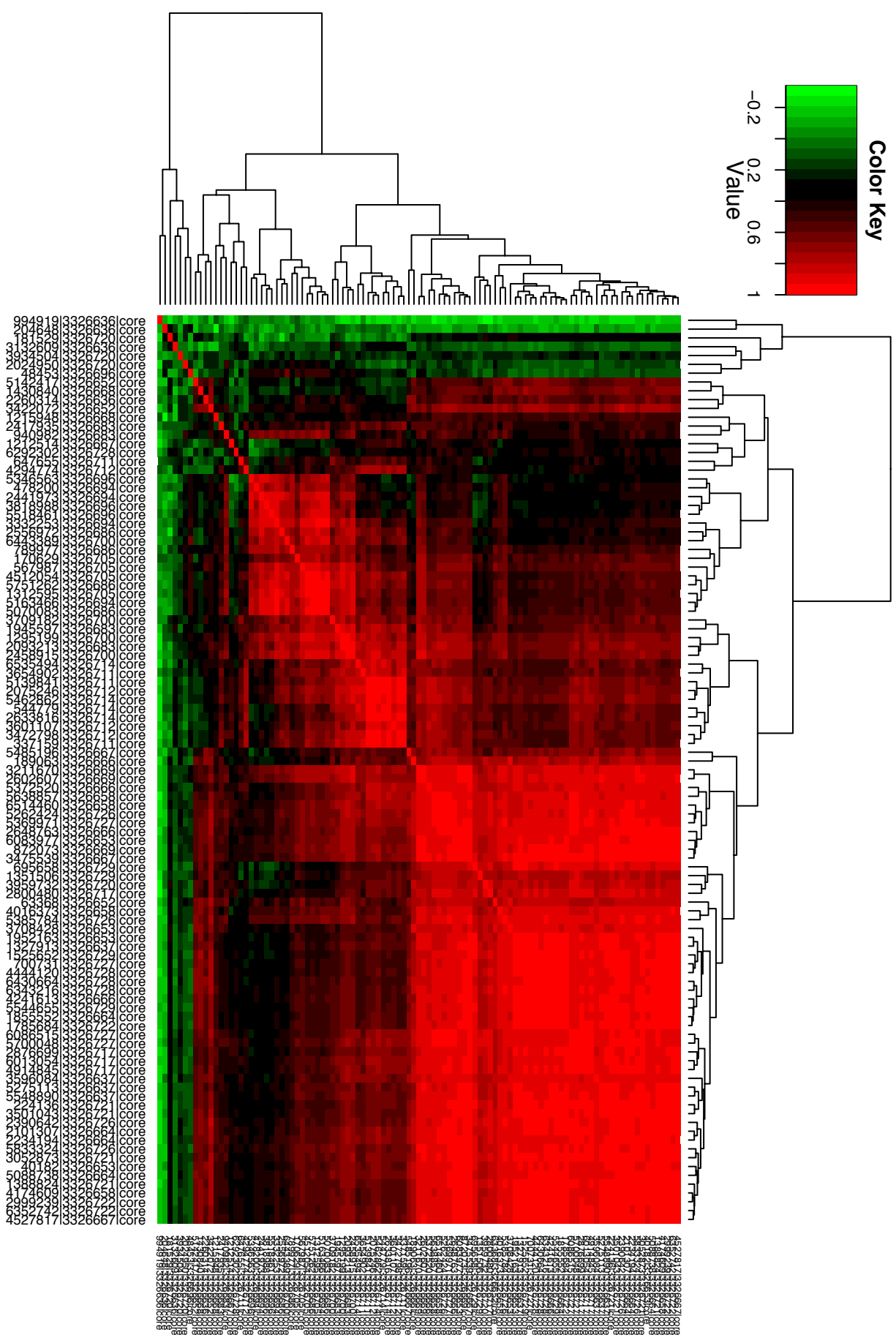

Supplement: Figure S2 — Heatmap visualization (high-resolution) of probe intensities of core probes in CD44 (transcript cluster 3326635). Probes targeting the 5' and 3' regions (constitutive exons) of CD44 show highly correlated signals in 11 tissues (the top right corner of the heatmap). (0.28 MB PDF) [file pone.0000088.s002.pdf]
