# Supplementary material for: Probe Selection and Expression Index Computation of Affymetrix Exon Arrays
Source: PLoS One. 2006 Dec 20;1(1):e88. doi: 10.1371/journal.pone.0000088 (PMC1762343; doi:10.1371/journal.pone.0000088)
Supplement: Figure S3 — Heatmap visualization (high-resolution) of probe intensities of core probes in CD44 (transcript cluster 3326635). Probes are ordered from top to bottom based on their genomic coordinates (5' to 3'). (0.05 MB PDF) [file pone.0000088.s003.pdf]

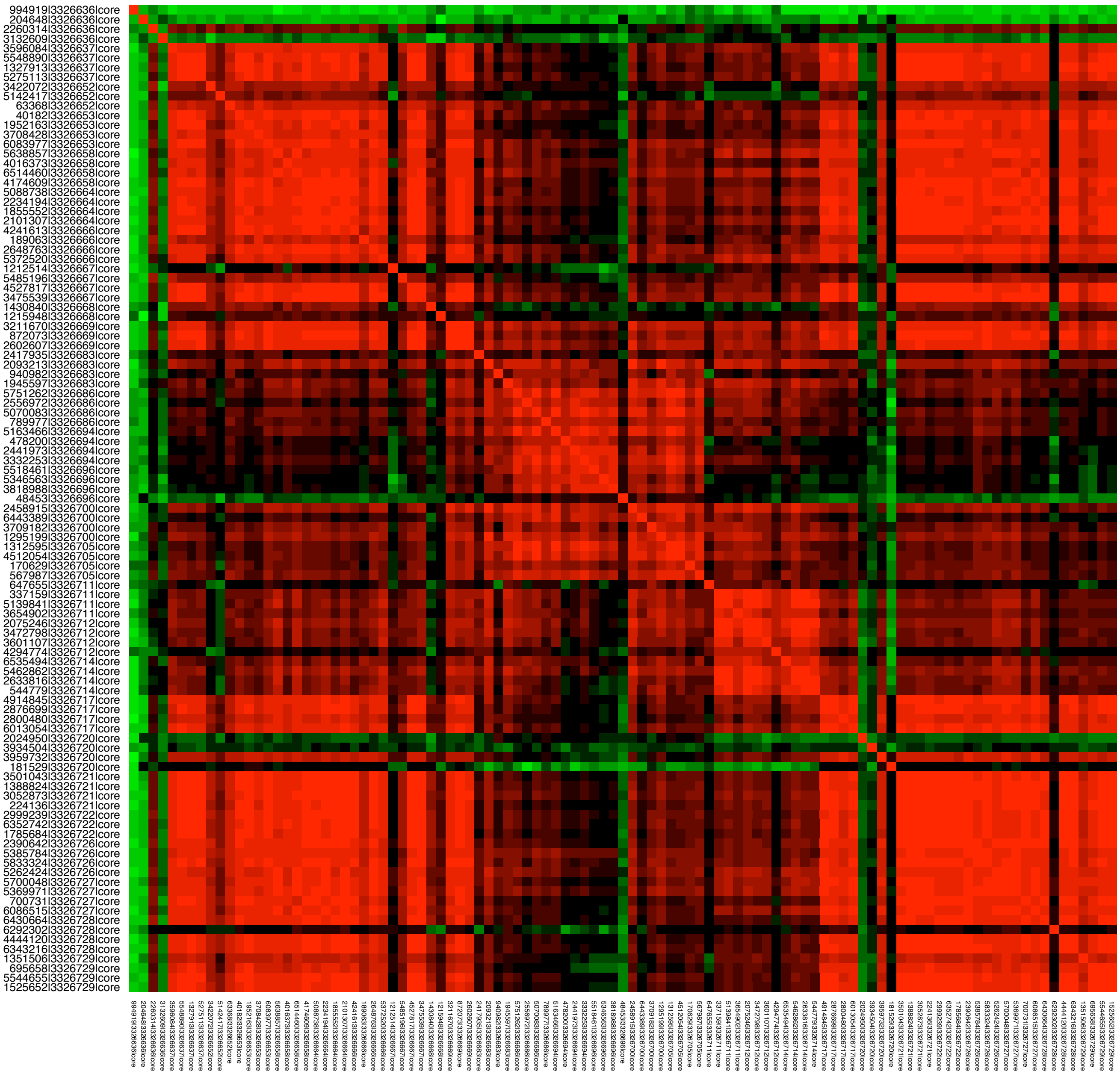
994919|3326636|core
204648|3326636|core
2260314|3326636|core
3132609|3326636|core
3596084|3326637|core
5548890|3326637|core
1327913|3326637|core
5275113|3326637|core
3422072|3326652|core
5142417|3326652|core
63368|3326652|core
40182|3326653|core
1952163|3326653|core
3708428|3326653|core
6083977|3326653|core
5638857|3326658|core
4016373|3326658|core
6514460|3326658|core
4174609|3326658|core
5088738|3326664|core
2234194|3326664|core
1855552|3326664|core
2101307|3326664|core
4241613|3326666|core
189063|3326666|core
2648763|3326666|core
5372520|3326666|core
1212514|3326667|core
5485196|3326667|core
4527817|3326667|core
3475539|3326667|core
1430840|3326668|core
1215948|3326668|core
3211670|3326669|core
872073|3326669|core
2602607|3326669|core
2417935|3326683|core
2093213|3326683|core
940982|3326683|core
1945597|3326683|core
5751262|3326686|core
2556972|3326686|core
5070083|3326686|core
789977|3326686|core
5163466|3326694|core
478200|3326694|core
2441973|3326694|core
3332253|3326694|core
5518461|3326696|core
5346563|3326696|core
3818988|3326696|core
48453|3326696|core
2458915|3326700|core
6443389|3326700|core
3709182|3326700|core
1295199|3326700|core
1312595|3326705|core
4512054|3326705|core
170629|3326705|core
567987|3326705|core
647655|3326711|core
337159|3326711|core
5139841|3326711|core
3654902|3326711|core
2075246|3326712|core
3472798|3326712|core
3601107|3326712|core
4294774|3326712|core
6535494|3326714|core
5462862|3326714|core
2633816|3326714|core
544779|3326714|core
4914845|3326717|core
2876699|3326717|core
2800480|3326717|core
6013054|3326717|core
2024950|3326720|core
3934504|3326720|core
3959732|3326720|core
181529|3326720|core
3501043|3326721|core
1388824|3326721|core
3052873|3326721|core
224136|3326721|core
2999239|3326722|core
6352742|3326722|core
1785684|3326722|core
2390642|3326726|core
5385784|3326726|core
5833324|3326726|core
5262424|3326726|core
5700048|3326727|core
5369971|3326727|core
700731|3326727|core
6086515|3326727|core
6430664|3326728|core
6292302|3326728|core
4444120|3326728|core
6343216|3326728|core
1351506|3326729|core
695658|3326729|core
5544655|3326729|core
1525652|3326729|core
